# Psychosis secondary to thyrotoxicosis that persisted post-thyroidectomy: a case report

**DOI:** 10.1186/s12888-023-05227-4

**Published:** 2023-10-13

**Authors:** Shiva Kothari, William Townsend, Zuhaib Chaudhry, Seth Kalin, Kevin Freeman

**Affiliations:** University of Mississippi Medical Center Jackson, MS 39216 Jackson, USA

**Keywords:** Thyrotoxicosis, Psychosis, Graves, Thyroidectomy, Antipsychotic

## Abstract

**Background:**

This case report is of a patient with psychosis secondary to thyrotoxicosis that persisted and reemerged after definitive treatment of thyroidectomy, which is a unique occurrence in the literature.

**Case presentation:**

This patient is a male between 30 and 35 years of age with a history of Graves Disease and no past psychiatric history who was admitted to the hospital due to psychosis secondary to thyrotoxicosis. The thyrotoxicosis was treated with surgical removal, but the psychotic symptoms persisted after surgery and normalization of standard thyroid functional measures. The symptoms were of sufficient significance for inpatient psychiatric hospitalization, a rare occurrence. Ultimately after an extended stay in the psychiatric unit, the patient’s symptoms stabilized with a second-generation antipsychotic, and the patient was discharged from the psychiatric unit.

**Conclusion:**

This case is evidence that the link between psychosis and hyperthyroidism is still poorly understood due to the patient’s psychotic symptoms persisting after the definitive treatment of thyroidectomy and the fact that it required anti-psychotic medications for normalization.

## Background

Psychosis, as defined by the Diagnostic Standard Manual V, is abnormalities in two or more of the following five domains of delusions, hallucinations, disorganized speech, grossly disorganized or abnormal behavior and negative symptoms (i.e. alogia, avolition or diminished emotional expression,) with one abnormality in the domain of delusion, hallucinations or disorganized speech [1]. The etiology of psychosis in patients is often multifactorial and most often seen in primary thought disorders such as schizophrenia or schizoaffective disorders [2]. Uncommonly, patients may also develop psychosis from medical conditions, which include thyroid abnormalities [3]. Thyrotoxicosis is defined as a state in which increased levels of circulating thyroid hormones are observed and is a state that can be life-threatening and requires prompt medical attention [4]. Thyrotoxicosis presenting with psychosis has been reported, though the cases are few [5]. Fewer still are cases in which psychiatric symptoms persist after thyrotoxicosis is treated [6]. However, most patients with acute psychiatric conditions post-treatment have underlying hypothyroid levels with seizures [8]. This underscores the rarity of this case, as there have been few to no reports of post-treatment acute psychosis with euthyroid levels requiring antipsychotic medications and extensive inpatient psychiatric admission.

## Case presentation

### Condition and history

In this case, a 30–35 year old African American male, with a past medical history of Graves Disease diagnosed in 2020 on methimazole and no past psychiatric history, presented to the emergency department (ED) with altered mental status (AMS). The patient could not provide history at the time of arrival due to AMS and disorganized behavior, so most history was initially provided by the patient’s mother. Per mother, the patient’s behavior over the prior two months was uncharacteristic, including posting threatening messages on social media, purchasing firearms, being incarcerated for stealing work property, and delusions such as believing he was a villain destined to kill superheroes. In addition, the patient had homicidal ideation towards family members, leading his mother to no longer feel safe in his presence. The mother reported a history of abnormal behavior a month before the patient’s diagnosis of Graves’ Disease in 2020 in which the patient was paranoid, anorexic, and had insomnia. At that time, the patient was stabilized with methimazole and had complied with his medication until two months before this admission.

### Examination, results and intensive care unit (ICU) stay

When the patient arrived at the ED, the patient was agitated, uncooperative, and combative, requiring restraints and sedation by a combination of Haloperidol (Haldol) 10 mg/ Midazolam (Versed) 2 mg intramuscular. Initially, the ED believed the patient’s symptoms were due to a brief psychotic episode or drug-related psychosis, so the patient was transferred to the psychiatric emergency unit. The urine drug screen was positive for cannabinoids with a false positive for benzodiazepines from the required sedation of Haldol 10 mg/Versed 2 mg IM. The patient denied any other history of illicit substance use and no previous, documented records of stimulant use disorder were identified. Overnight, the patient had persistent tachycardia of > 110 heart rate, so a thyroid panel was initiated, which resulted in Thyroid Stimulating Hormone (TSH) < 0.0005 and free T_4_ (fT4) > 7.770, evidence for a thyrotoxicosis induced psychosis. Other labs included a complete blood count and a complete metabolic panel, which were benign.

The patient was admitted to the Intensive Care Unit (ICU) to treat his thyrotoxicosis. While in the ICU, the patient reported paranoia, and persecutory delusions causing distress due to concerns that he was the subject of the medical staff’s discussions in the hallway. Nursing reported that the patient was concerned that the medical staff had malicious intentions, so psychiatry started the patient on Olanzapine 10 mg twice a day (BID). Endocrine was consulted and initiated Propranolol 60 mg three times a day (TID) and Methimazole 30 mg BID and noted thyromegaly and peripheral tremors on physical exam. During the two days in the ICU, the patient’s thyroid levels improved with decreased paranoia and combativeness, and he was transferred to the hospital’s internal medicine floor for continued monitoring. The endocrine team recommended a thyroidectomy due to his recent history of noncompliance and the high mortality risk of thyrotoxicosis. Once the patient had a normal fT4 level of 2.27ng/dL, otolaryngology performed a total thyroidectomy, and the patient was transferred back to the inpatient hospital floor for continued monitoring. With no evidence of psychosis and overall improvement in behavior on postoperative day (POD) 1, Olanzapine 10 mg BID was decreased to once nightly.

### Psychiatric management and inpatient psychiatry unit stay

On POD 6, the patient’s paranoia towards hospital staff returned even with reassuring labs of parathyroid hormones (PTH) 41.3pg/ml and calcium 8.9mmol/L, good adherence to Levothyroxine (Synthroid) 137 mcg daily, and a benign physical exam. The patient was then transferred to the inpatient psychiatric unit and diagnosed with a psychotic disorder due to hyperthyroidism, with delusional disorder. The patient continued Synthroid 137 mcg/daily in the psychiatric unit and restarted Olanzapine 10 mg BID. Olanzapine 10 mg BID was down-titrated to 10 mg nightly for better patient compliance with a simplified regimen and to avoid oversedation from higher doses of an antipsychotic. After day two on the psychiatric floor, the patient’s psychosis symptoms abated on the antipsychotic. Upon questioning, the patient had no recollection of the past two months, including most of his hospital stay. Ultimately, the patient showed greater insight into his illness and understood the need for strong medical compliance and outpatient follow-up. The patient was discharged on Synthroid 137 mcg daily and Olanzapine 10 mg daily. Post hospitalization, the patient was lost to follow up.

## Discussion and conclusions

The current case report describes a 30-35-year-old African American male with no past psychiatric history who presented with psychotic-like symptoms and was ultimately found to have psychotic disorder due to hyperthyroidism. The hyperthyroidism was treated with surgical removal, but uniquely, the psychotic symptoms persisted post-operatively even with evidence of euthyroid, normal PTH and calcium levels. The symptoms were significant enough to lead to multiple-day inpatient psychiatric hospitalization. Ultimately, the patient was treated with a second-generation antipsychotic Olanzapine, resolving the psychotic symptoms.

### Psychiatric condition and beta-adrenergic activation in hyperthyroidism

Although the overlap between symptoms of hyperthyroidism and psychiatric conditions is significant, vital signs and physical exam abnormalities often can distinguish between the two. Persistent tachycardia can distinguish between psychiatric conditions such as mania or psychosis and hyperthyroidism. However, one must use caution to use those objective measures, as tachycardia can often exist in co-morbid psychiatric conditions such as anxiety, substance use, or several other medical conditions (6; 9). In hyperthyroidism, there is an overactivation of beta-adrenergic receptor mediation of catecholamines, which could cause agitation, aggression, and tachycardia [3]. In our case, the patient had benign vital sign parameters initially, with no tachycardia. He later developed tachycardia which led to thyroid testing, so vital signs were useful in formulating the differential diagnosis.

### Comparison of current case to past cases of psychosis secondary to thyrotoxicosis

Previous case reports have shown that psychosis secondary to thyrotoxicosis have multiple primary psychiatric etiologies including depression and mania [5]. Our patient had unknown etiology of psychosis, but our leading differentials were related to medical condition (thyrotoxicosis), primary thought disorder such as unspecified schizophrenia spectrum or other psychotic disorder or due to substance-induced psychotic disorder secondary to cannabis use. Interestingly, for psychosis secondary to thyrotoxicosis, most case reports have a majority of non-primary thought disorders as etiology [5].

### Pathogenesis of thyrotoxicosis-induced psychosis

There is a prevalence of data that suggests beta-adrenergic activation would help explain the similarity of thyrotoxicosis to symptoms such as mania, aggression or anxiety (6;9). However, the connection of thyrotoxicosis to psychosis is less clear. It is thought that, in Graves Disease, the increase in TSH receptor (TSHR) antibodies activates TSH receptors in the hippocampus and cerebrum, producing neuropsychiatric symptoms [14]. In addition, studies have shown altered glucose metabolism in limbic structures in patients with hyperthyroid disease states implicating the limbic system in the manifestation of psychiatric symptoms [14, 15]. Psychosis brain imaging studies indicate that the frontal lobe and limbic lobe are impacted in schizophrenia perhaps further explaining the link [5, 9, 10].

### Treatment

Treating thyrotoxicosis-induced psychosis typically involves a combination of medical and psychiatric treatment. The primary goal is to reduce the thyroid hormone levels to normal ranges with antithyroid medications such as Methimazole and Propyluracil, but those effects may not be seen for several weeks. Therefore, more invasive treatments could be necessary such as thyroidectomy or radioactive iodine ablation [7]. Symptomatically, beta-blockers have a unique role in treatment of thyrotoxicosis induced psychosis as they are indicated for thyrotoxicosis treatment but also indicated for treatment of anxiety, a psychiatric illness. They provide a dual role in their indication. Psychiatric evaluation is recommended in these patients to rule out organic psychiatric illness like bipolar disorder or anxiety or panic attacks. In terms of psychiatric medications, if psychotic symptoms persist, second generation antipsychotics such as Risperidone or Olanzapine may be indicated. First generation antipsychotic could help but there are case reports of Haldol causing dystonia and even thyroid storm in some patients [16]. Lithium is a unique medication in that it is approved by the U.S Food and Drug Administration for treatment of acute mania and has well-known antithyroid properties. Ultimately, patients should have psychiatric and medical outpatient follow ups to monitor for signs of psychosis and thyroid levels.

### Significance

This case presentation is one of many reports displaying psychosis due to hyperthyroidism. However, there are few reports of patients having two instances of acute psychosis in one hospital admission with one instance of psychosis being POD6 of a thyroidectomy (5; 7; 9). In terms of medication, olanzapine’s mechanism of action of antagonizing dopamine D2 receptors in the mesolimbic pathway could explain the decrease in delusions and disorganized behavior once this medication was started [12].

When patients present to the ED with psychosis, it is important to have hyperthyroidism on the differential and to order TSH/triiodothyronine (T3)/fT4 labs. Thyrotoxicosis’ high mortality rate of 8–25% increases the urgency of immediate treatment with antithyroid medications. On physical examination, persistent tachycardia and thyromegaly are typical findings guiding workup toward thyrotoxicosis-induced psychosis. One must use caution in using objective measures alone, as tachycardia can often exist in co-morbid psychiatric conditions such as anxiety and substance use, displaying the importance of ordering thyroid labs.

This case’s acute psychosis after thyroidectomy is not fully understood, and more research on Graves’ Disease complications post-thyroidectomy needs to be performed to understand the etiology of this rebound acute psychosis. However, this case displays the importance of medication compliance with antithyroid medication in Graves Disease patients. This patient’s ED presentation could have been averted if he was compliant with the prescribed Methimazole. This case demonstrates the need for extended monitoring of Graves Disease patients post-thyroidectomy. This patient was asymptomatic POD1-4, but POD5, the patient redeveloped delusional thinking and combativeness. The patient could have developed these neuropsychiatric issues at home with the inability to adhere to his Synthroid and Olanzapine medications. Close follow-ups are needed in these patients to monitor TSH/T3/fT4 labs and assess instances of psychosis or psychiatric abnormalities.

### Limitations and Future Studies

Limitations of this case include lack of long-term follow up to see how long the symptoms of psychosis persisted and if the anti-psychotic effectively treated those symptoms. It is unclear if the patient has an underlying primary thought disorder that was the actual etiology of the psychotic symptoms after removal of the thyroid. Due to the diagnostic time frame and clinical course of primary thought disorders like schizophrenia and schizoaffective disorder, it can often take years before an accurate diagnosis can be achieved. Future studies can explore the true prevalence of psychosis due to thyrotoxicosis or discover the true incidence of psychosis persisting even after treatment of medical conditions that cause psychosis.

## Data Availability

Not Applicable.

## Abbreviations

AMS: Altered Mental Status
ED: Emergency Department
fT4: Free-Thyroxine
Haldol: Haloperidol
ICU: Intensive Care Unit
Synthroid: Levothyroxine
Versed: Midazolam
POD: Postoperative Day
PTH: Parathyroid Hormone
TID: Three Times a Day
TSHR: Thyroid Stimulating Hormone Receptor
TSH: Thyroid Stimulating Hormone
T3: Triiodothyronine
BID: Twice a Day
